# UPLC-Q-TOF-MS-based unbiased serum metabolomics investigation of cholangiocarcinoma

**DOI:** 10.3389/fmolb.2025.1549223

**Published:** 2025-04-07

**Authors:** Xiaowei Wang, Xuefeng Xu, Ran Jia, Yuanhong Xu, Ping Hu

**Affiliations:** ^1^ Department of Clinical Laboratory, The First Affiliated Hospital of Anhui Medical University, Hefei, China; ^2^ Department of Hepatobiliary surgery, The First Affiliated Hospital of Anhui Medical University, Hefei, China; ^3^ School of Chemistry and Molecular Engineering, East China University of Science and Technology, Shanghai, China

**Keywords:** cholangiocarcinoma, diagnosis, unbiased serum metabolomics, UPLC-Q-ToF-MS, biomarker screen and identification

## Abstract

**Objective:**

Cholangiocarcinoma (CCA) is a highly aggressive malignancy, and early diagnosis remains challenging. Metabolic biomarkers are increasingly recognized as promising tools for the early detection of cancer. However, a comprehensive exploration of metabolic alterations in CCA, especially from a global metabolic perspective, has yet to be fully realized. To identify reliable metabolic markers for the early diagnosis of CCA and to explore its potential pathogenesis through an in-depth analysis of global metabolism.

**Methods:**

Serum samples from 30 CCA patients and 31 healthy individuals were analyzed using an unbiased UPLC-Q-TOF-MS based metabolomics approach. Principal component analysis (PCA) and orthogonal projections to latent structures discriminant analysis (OPLS-DA) were applied to identify potential biomarkers. High-resolution MS/MS and available standards were used to further confirm the identified metabolites. A systematic metabolic pathway analysis was conducted to interpret the biological roles of these biomarkers and explore their relevance to CCA progression.

**Results:**

A total of 25 marker metabolites were identified, including lysophosphatidylcholines (LysoPCs), phosphatidylcholines (PCs), organic acids, sphinganine, and ketoleucine. These metabolites effectively distinguished CCA patients from healthy controls, with an AUC of 0.995 for increased biomarkers and 0.992 for decreased biomarkers in positive mode. In negative mode, the AUC for increased and decreased biomarkers was 0.899 and 0.976, respectively. The metabolic pathway analysis revealed critical biological functions linked to these biomarkers, offering insights into the molecular mechanisms underlying CCA initiation and progression.

**Conclusion:**

This study identifies novel metabolic biomarkers for the early diagnosis of CCA and provides a deeper understanding of the metabolic alterations associated with the disease. These findings could contribute to the development of diagnostic strategies and therapeutic interventions for CCA.

## 1 Introduction

Cholangiocarcinoma (CCA) is a rare and heterogeneous malignancy of the biliary system, originating from the epithelial cells lining the bile duct (Banales et al., 2020). CCA is currently the second most prevalent primary liver cancer after hepatocellular carcinoma (Banales et al., 2016) and is classified into three subtypes based on anatomical location: intrahepatic (iCCA), perihilar (pCCA), and distal (dCCA) (Sulpice et al., 2013). Although CCA remains relatively uncommon, its clinical incidence has been steadily increasing over recent decades, reaching an annual rate of 0.3–6 cases per 100,000 individuals (Guilhamon et al., 2013). Traditional diagnostic methods are hindered by a lack of sensitive and specific tests. While magnetic resonance imaging, computed tomography, and endoscopic ultrasound offer useful diagnostic insights in select patients, these techniques are often invasive, time-consuming, and costly. Early diagnosis remains challenging, with ultrasonography and endoscopic retrograde cholangiography still being the primary tools for CCA detection (Intuyod et al., 2019), yet these methods often fail to identify the tumor at its earliest stages, contributing to the disease’s high mortality rates (Wang et al., 2013; Lindner et al., 2015). Although nonspecific biomarkers such as carbohydrate antigen 199 (CA199) (Liang et al., 2015) and carcinoembryonic antigen (CEA) (Loosen et al., 2017) are used in diagnosis, their levels are also elevated in hepatocellular carcinoma, cholangitis, and other hepatobiliary diseases, complicating their reliability as diagnostic markers for CCA (Macias et al., 2018; Chen et al., 2002). Therefore, there is an urgent need for the identification of specific biomarkers to facilitate early detection, improve prognosis, and guide treatment strategies for CCA.

Metabolomics, a powerful analytical approach developed after genomics, transcriptomics, and proteomics (Saito and Matsuda, 2010), is emerging as a promising tool for biomarker discovery. By qualitatively and quantitatively analyzing small-molecule endogenous metabolites in biological samples, metabolomics identifies correlations between metabolic alterations and pathological changes (Suhre et al., 2011). This technique allows for comprehensive analysis of metabolites in various biological matrices, such as serum, urine, feces, and tissues, to pinpoint disease-specific biomarkers (Chua et al., 2013). It offers the advantage of detecting subtle biomarker changes, providing early warnings of disease onset, and reflecting the progression and severity of the disease (Pintus et al., 2017). Consequently, metabolomics is particularly well-suited for tumor diagnosis (Yang et al., 2018), biomarker discovery (Burton and Ma, 2019), and prognostic evaluation (Buck et al., 2017), owing to its high-throughput, systematic, and holistic nature. Recent studies have demonstrated the utility of metabolomics in CCA. Alsaleh et al. identified distinct metabolic profiles in urinary samples from healthy controls versus individuals with CCA, with altered acylcarnitine levels identified through orthogonal projections to latent structures discriminant analysis (OPLS-DA) (Alsaleh et al., 2019). Among them, ultra-performance liquid chromatography coupled with quadrupole time-of-flight mass spectrometry (UPLC-Q-TOF-MS) offers high sensitivity, resolution, and accuracy in detecting a broad range of metabolites in biological samples. The ability of UPLC-Q-TOF-MS to comprehensively profile metabolic changes makes it particularly suitable for identifying potential biomarkers for CCA from blood samples, which are easily accessible and minimally invasive compared to tissue or bile specimens. They also observed changes in the abundance of phospholipids in serum metabolites, particularly in patients with liver disease, including CCA, as detected by unsupervised principal component analysis (PCA), although no significant differences were found between profiles from CCA patients and those with benign biliary strictures (Alsaleh et al., 2020). Additionally, Banales et al. proposed a combined PCA and OPLS analysis to differentiate between iCCA and hepatocellular carcinoma (HCC), enabling accurate tumor classification based on biopsy-proven diagnoses (Banales et al., 2019).

In this study, serum metabolite profiles of CCA patients and healthy controls were analyzed using ultraperformance liquid chromatography-quadrupole-time-of-flight mass spectrometry (UPLC-Q-TOF-MS) based metabolomics. Serum was chosen over urine as the biological sample of interest because clinical urine samples require 24-hour collection periods for reliable metabolite quantification, resulting in more complex postprocessing (Ren et al., 2021). Furthermore, serum provides a more representative snapshot of metabolic activity and offers richer chemical information, making it a preferred sample type in metabolomics research (Sun et al., 2013). Through systematic statistical analysis, this study aims to gain insights into the metabolic alterations associated with CCA, advancing the identification of specific biomarkers for early diagnosis and enhancing our understanding of disease progression at the metabolic level.

## 2 Materials and methods

### 2.1 Reagents and chemicals

Acetonitrile (ACN, LC-MS grade) and methanol (LC-MS grade) were procured from Merck (Darmstadt, Germany). Formic acid (FA, LC-MS grade) was sourced from ACS (Anaqua Chemicals Supply, United States), and ultrapure water used in this study was obtained via an EPED-E2-10 TF system (Nanjing, China). The lysophosphatidylcholine (LysoPC) standard, LysoPC(16:0/0:0), was obtained from AvantiPolar Lipids (Alabaster, AL, United States). Other standards, including D-glucuronic acid, 2-hydroxybutyric acid, ketoleucine, alpha-ketoisovaleric acid, and glyceric acid, were supplied by Shanghai Macklin Biochemical Technology Co., Ltd. (Shanghai, China).

### 2.2 Clinical sample collection

This study enrolled CCA patients from the First Affiliated Hospital of Anhui Medical University between October 2020 and March 2021, while healthy controls were recruited from medical examiners. The study utilized discarded specimens, originally collected for clinical purposes and deemed surplus to diagnostic or treatment procedures, for analysis. These specimens posed no additional risk to participants, as strict ethical guidelines and data protection protocols were followed during anonymization and handling. Ethical approval (Approval No.: PJ2024-11-35) was granted, ensuring compliance with ethical standards and safeguarding participant confidentiality. CCA patients included in the study had been clinically diagnosed with CCA for at least 6 months and were confirmed by two independent pathologists. The healthy control group was matched to the CCA patients by age and gender. All physiological indicators were derived from 8-hour fasting blood samples (Table 1), which included: (i) liver function indicators such as ALT, AST, ALP, GGT, TBIL, DBIL, and UREA, and (ii) tumor markers, including AFP, CEA, CA-125, and CA199.

**TABLE 1:** Demographic and clinical characteristics of CCA patients.

Characteristics	Control (n = 31)	CCA (n = 30)
Sex (F/M)	12/19	11/19
Age, years	56 ± 7	63 ± 10
Age, range	50–85	47–89
ALT (IU/L)	26.00 ± 10.21	150.62 ± 182.35
AST (IU/L)	20.42 ± 6.18	106.03 ± 118.45
ALP (IU/L)	78.80 ± 21.21	351.40 ± 279.26
GGT (IU/L)	28.20 ± 24.84	421.40 ± 500.29
TBIL (mg/dL)	14.18 ± 4.43	157.78 ± 148.29
DBIL (mg/dL)	4.61 ± 1.53	270.29 ± 745.84
Urea (ng/mL)	5.08 ± 1.13	8.90 ± 14.97
AFP (ng/mL)	3.02 ± 1.77	3.21 ± 2.43
CEA (ng/mL)	1.87 ± 1.10	7.28 ± 7.38
CA-125 (IU/mL)	11.8 ± 5.0	46.40 ± 72.17
CA-199 (IU/mL)	10.37 ± 5.77	876.65 ± 1,474.62

### 2.3 Sample preparation

Frozen serum samples were thawed on ice, and 100 μL of serum was mixed with 400 μL of ACN, followed by vortexing for 1 min. The samples were then centrifuged at 13,000 rpm for 10 min to remove proteins. The resulting supernatant was aliquoted into 400 μL portions and freeze-dried. After freeze-drying, the product was reconstituted in 100 μL of H_2_O/ACN and centrifuged at 13,000 rpm for 10 min at 4°C. The supernatant was then filtered through a 0.22-μm-thick GHP membrane (PALL Corporation, United States) prior to UPLC-MS analysis.

### 2.4 UPLC-Q-TOF-MS condition

Chromatographic separation was carried out using an Agilent 1290 UPLC system with an Acquity BEH C18 column (100 mm × 2.1 mm, 1.7 μm) at a column temperature of 45°C. The injection volume was set to 5 μL, and the flow rate was fixed at 0.5 mL/min. A gradient mobile phase was employed, and the time schedule is provided in Supplementary Table S1. Mobile phase A consisted of 0.1% FA in H_2_O, and mobile phase B was 0.1% FA in ACN. MS detection was performed using a Q-TOF mass spectrometer with the following settings: drying gas temperature at 350°C; drying gas flow at 10 L/min; nebulizer pressure at 30 psig; capillary voltage set at 3,500 V for positive mode and 3,000 V for negative mode; fragmentor voltage at 125 V; skimmer voltage at 65 V; and octopole RF voltage at 750 V. The collision energies for targeted MS/MS analysis were set at 20 and 40 eV, respectively. Mass spectra were acquired in the m/z range of 50–1,200, with a scan rate of one spectrum per second. For accurate mass measurement, continuous calibration was performed using reference solutions with reference masses of *m/z* 121.0509 and 922.0098 (positive mode) or *m/z* 112.9856 and 1,033.9881 (negative mode).

### 2.5 Data processing and analysis

OPLS-DA was employed as a common method to identify potential marker metabolites in serum. In this study, metabolites were considered markers if their variable importance in projection (VIP) score exceeded 1.0 and their P-value was less than 0.05, with a *t*-test used to assess reliability. The total ion chromatographic data obtained from Q-TOF analysis were converted into. mzML files using ProteoWizard software and processed with Progenesis QI v2.0 software (Waters, Newcastle, United Kingdom). Representative sample data were selected for automatic alignment with other datasets using Progenesis QI. Following this, adduct ions were deconvoluted, and ion abundance was calculated based on a threshold level. All detected features were matched against a serum metabolite database, using a mass tolerance of 10 ppm (serum metabolite online database, https://hmdb.ca/). Raw data obtained from UPLC-Q-TOF-MS were first processed for noise reduction and baseline correction to improve data quality. Then, peak alignment and integration were carried out to quantify the compounds. To ensure comparability of data across samples, normalization methods like mean normalization or total ion count (TIC) normalization were applied. Scaling was also performed using approaches such as standardization (Z - score) and min - max scaling, which helped to avoid the dominance of variables with different units or scales in the analysis. Heatmap was plotted by https://www.bioinformatics.com.cn (last accessed on 10 December 2024), an online platform for data analysis and visualization. Receiver operating characteristic (ROC) analysis was performed using SPSS 24.0 software to evaluate the accuracy of the statistical results. Additionally, differential metabolites were analyzed for pathway enrichment using MetaboAnalyst, and the metabolite pathway network was visualized using MetScape.The criteria for selecting marker metabolites in both groups included a VIP value greater than 1.0 and a P-value less than 0.05.

## 3 Results

Initially, we conducted a retrospective analysis of patient clinical data and subsequently present the fundamental characteristics of the collected samples in Table 1. The results indicate that there were no statistically significant differences observed in the variables of gender. However, a significant difference was found in levels alanine aminotransferase (ALT), and alanine aminotransferase (AST), alkaline phosphatase (ALP), gamma glutamyl transferase (GGT), total bilirubin (TBIL), direct bilirubin (DBIL), Urea, AFP, CEA and CA-199 between Control and CCA samples. between the two groups.

### 3.1 PCA of CCA and normal sera

**FIGURE 1:**
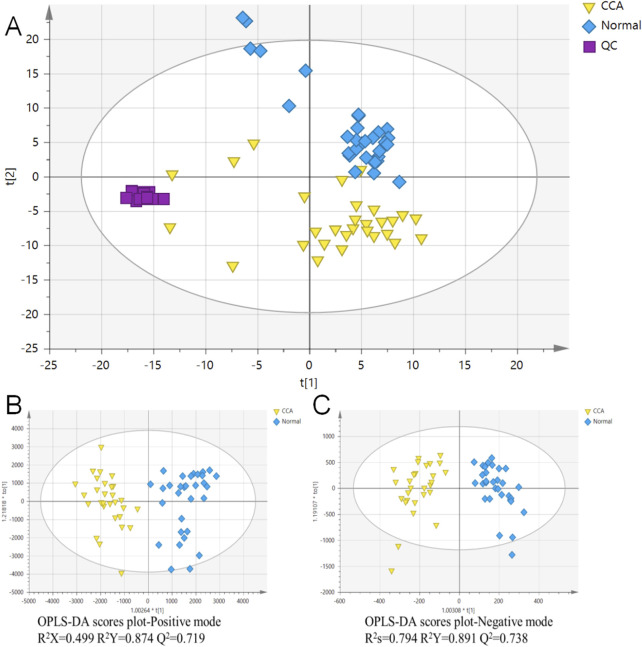
Principal Component Analysis (PCA) and Orthogonal Partial Least Squares Discriminant Analysis (OPLS-DA) of cholangiocarcinoma (CCA) and normal serum samples. **(A)** PCA analysis was conducted on combined data to visualize the overall distribution of CCA and normal serum samples. **(B)** OPLS-DA analysis of CCA and normal serum samples in the positive ion mode. **(C)** OPLS-DA analysis of CCA and normal serum samples in the negative ion mode. The quality control (QC) samples are indicated for quality assurance in the analysis. CCA: cholangiocarcinoma; QC: quality control; n = 30 for CCA and n = 31 for normal serum samples.

In this study, total ion chromatograms were collected in both positive and negative ion modes for normal and CCA samples, and a nontargeted metabolic approach was applied. High-quality data are essential for robust metabolomics analysis. Quality control (QC) samples were included to assess the repeatability and stability of the analytical method. The PCA score plot (Figure 1A) showed tight clustering of the QC samples, indicating the reliability of the experimental setup. Clear separation was observed between the diseased and normal groups, suggesting that CCA significantly disrupts the metabolic profile in the serum of affected patients. Furthermore, OPLS-DA was used for supervised analysis of group differences, as shown in Figures 1B,C. Both positive and negative ion mode scores indicated that this method was stable and had strong predictive capability. Predicted candidate biomarkers were identified and listed in Table 2 (16 candidate biomarkers in positive mode, nine in negative mode).

**TABLE 2:** Discriminant serum metabolites between CCA and normal samples.

No.	RT (min)	*m/z*	Adducts	Formula	Description	MS/MS fragments	Trend	VIP
1	9.96	520.3399	M + H	C_26_H_50_NO_7_P	LysoPC (18:2/0:0)	258.1085 [M-FA18:2 + H]^+^, 184.0728 [phosphocholine]^+^, 104.1071 [choline]^+^	↑	12.45
2	10.23	714.5409	M + H	C_40_H_76_NO_7_P	PC (P-18:1/14:1)	506.3520 [M-FA14:1 + H]^+^, 450.2861 [M-(P-18:1) + H]^+^ 184.0717 [phosphocholine]^+^, 104.1088 [choline]^+^	↓	3.89
3	10.18	518.3227	M + H	C_26_H_48_NO_7_P	LysoPC (18:3/0:0)	258.1104 [M-FA18:3 + H]^+^, 184.0725 [phosphocholine]^+^, 104.1067 [choline]^+^	↑	3.82
4	9.90	568.3413	M + H	C_30_H_50_NO_7_P	LysoPC (22:6/0:0)	184.0731 [phosphocholine]^+^, 104.1064 [choline]^+^	↑	3.48
5	11.70	546.355	M + H	C_28_H_52_NO_7_P	LysoPC (20:3/0:0)	184.0720 [phosphocholine]^+^, 104.1065 [choline]^+^	↑	3.35
6	10.38	480.3442	M + H	C_24_H_50_NO_6_P	LysoPC (P-16:0/0:0)	258.1121 [M-(P-16:0)+H]^+^, 184.0728 [phosphocholine]^+^, 104.1077 [choline]^+^	↓	3.02
7	9.90	494.3264	M + H	C_24_H_48_NO_7_P	LysoPC (16:1/0:0)	258.1106 [M-FA16:1 + H]^+^, 184.0723 [phosphocholine]^+^, 104.1067 [choline]^+^	↑	2.80
8	9.83	542.3254	M + H	C_28_H_48_NO_7_P	LysoPC (20:5/0:0)	258.1064 [M-FA20:5 + H]^+^, 184.0739 [phosphocholine]^+^, 104.1067 [choline]^+^	↑	2.74
9^a^	11.71	496.3409	M + H	C_24_H_50_NO_7_P	LysoPC (16:0/0:0)	258.1097 [M-FA16:0 + H]^+^, 184.0728 [phosphocholine]^+^, 104.1069 [choline]^+^	↑	2.19
10^a^	8.35	466.3163	M + H	C_26_H_43_NO_6_	Glycohyocholic acid	448.3075 [M-H_2_O + H]^+^, 430.2976 [M-2H_2_O + H]^+^ 412.2844 [M-3H_2_O + H]^+^	↑	2.12
11^a^	9.70	302.3062	M + H	C_18_H_39_NO_2_	Sphinganine	284.2939 [M-H_2_O + H]^+^	↓	2.11
12	11.71	522.3555	M + H	C_26_H_52_NO_7_P	LysoPC (18:1/0:0)	258.1102 [M-FA16:1 + H]^+^, 184.0730 [phosphocholine]^+^, 104.1073 [choline]^+^	↑	1.98
13	11.71	508.3763	M + H	C_26_H_54_NO_6_P	LysoPC (P-18:0/0:0)	258.1120 [M-(P-18:0)^+^H]^+^, 184.0731 [phosphocholine]^+^, 104.1074 [choline]^+^	↓	1.59
14	10.41	506.3606	M + H	C_26_H_52_NO_6_P	LysoPC (P-18:1/0:0)	184.0721 [phosphocholine]^+^, 104.1081 [choline]^+^	↓	1.46
15	11.68	808.5839	M + H	C_46_H_82_NO_8_P	PC (18:1/20:4)	544.3319 [M-FA18:1 + H]^+^, 522.3462 [M-FA20:4 + H]^+^ 184.0751 [phosphocholine]^+^, 104.1113 [choline]^+^	↓	1.35
16	10.43	548.3725	M + H	C_28_H_54_NO_7_P	LysoPC (20:2/0:0)	184.0731 [phosphocholine]^+^, 104.1083 [choline]^+^	↑	1.15
17	7.15	514.2942	M − H	C_26_H_46_NO_7_P	LysoPC (18:4/0:0)	499.2749 [M-CH_3_-H]^-^, 257.1945 [FA18:4-H]^-^	↑	5.83
18^a^	0.52	193.0362	M − H	C_6_H_10_O_7_	D-glucuronic acid	149.0457 [M-CO_2_-H]^-^	↑	3.11
19^a^	0.97	103.0402	M − H	C_4_H_8_O_3_	2-Hydroxybutyric acid	84.0217 [M-H_2_O-H]^-^, 58.0425 [M-CO_2_-H]^-^	↑	3.04
20^a^	3.10	129.0559	M − H	C_6_H_10_O_3_	Ketoleucine	114.0342 [M-CH_3_-H]^-^, 85.0688 [M-CO_2_-H]^-^	↓	2.76
21	2.40	117.0558	M − H	C_5_H_10_O_3_	2-Hydroxy-3-methylbutyric acid	102.0330 [M-CH_3_-H]^-^, 99.0413 [M-H_2_O-H]^-^ 73.0644 [M-CO_2_-H]^-^	↑	1.67
22	0.52	145.0623	M − H	C_5_H_10_N_2_O_3_	Ureidoisobutyric acid	130.0383 [M-CH_3_-H]^-^, 85.0290 [M-(NH_2_)_2_CO-H]^-^	↑	1.23
23^a^	1.62	115.0397	M − H	C_5_H_8_O_3_	alpha-Ketoisovaleric acid	100.0152 [M-CH_3_-H]^-^, 71.0488 [M-CO_2_-H]^-^	↓	1.21
24	9.72	764.5619	M − H	C_44_H_80_NO_7_P	PC(O-16:1/20:4)	749.5405 [M-CH_3_-H]^-^, 303.2342 [FA20:4-H]^-^	↓	1.09
25^a^	0.57	105.0195	M − H	C_3_H_6_O_4_	Glyceric acid	87.0098 [M-H_2_O-H]^-^, 61.0300 [M-CO_2_-H]^-^	↓	1.08

LysoPC: lysophosphatidylcholine; PC: phosphatidylcholine.

^a^ Identification of the compound was confirmed by its pure standard. “↑” means a significantly higher level of metabolites in the CCA, group than in the normal group, whereas “↓”represents a significantly lower level of metabolites.

### 3.2 Metabolite disorder in serum with CCA

**FIGURE 2:**
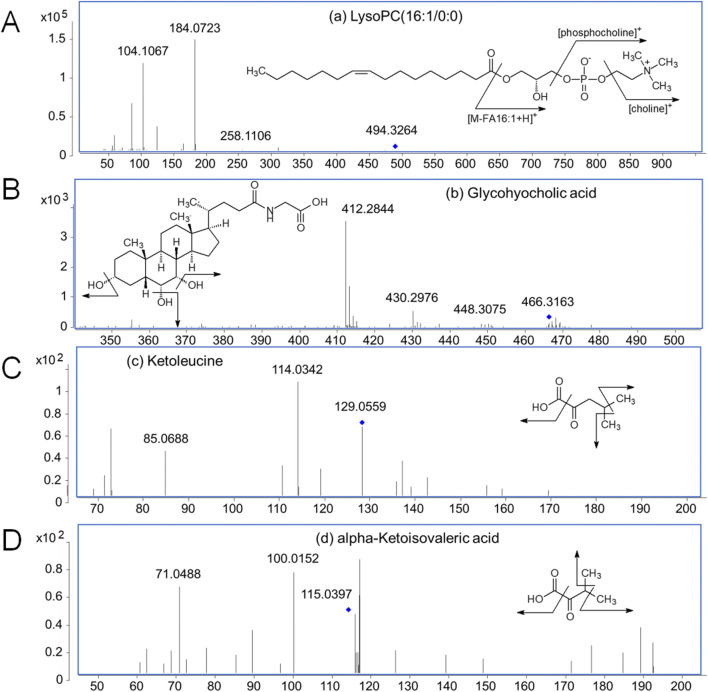
MS/MS spectra of four representative marker metabolites. **(A)** LysoPC (16:1/0:0) (No. 7 in Table 2); **(B)** Glycohyocholic acid (No. 10 in Table 2); **(C)** Ketoleucine (No. 20 in Table 2); **(D)** alpha-Ketoisovaleric acid (No. 23 in Table 2).

The serum differential metabolites were identified by matching their exact molecular masses and MS/MS spectra with the HMDB database (Figure 2). As examples, the fragmentation patterns of four representative marker metabolites are summarized in Figure 2. The abundant phosphocholine ion at *m/z* 184.0726 and choline ion at *m/z* 104.1067 are diagnostic fragments used to confirm the presence of phosphatidylcholine. The characteristic fragment ion at *m/z* 258.1106, generated by the cleavage of a fatty acyl chain, suggests the loss of the FA 16:1 group in LysoPC (16:1/0:0; No. 7). The successive neutral loss of H_2_O observed in the MS/MS spectra corresponds to the hydroxyl group in glycohyocholic acid (No. 10). For ketoleucine (No. 20) and alpha-ketoisovaleric acid (No. 23), the sequential neutral loss of CH_3_ and CO_2_ indicates their methyl and carboxyl structures. Based on these fragmentation patterns, a total of 25 marker metabolites were identified (Table 2).

**FIGURE 3:**
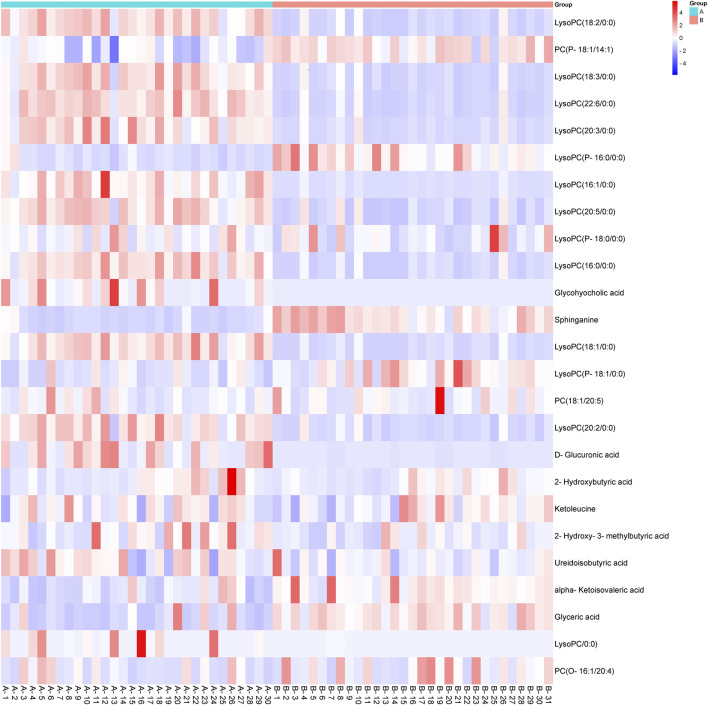
Heatmap showing the metabolic profiling of cholangiocarcinoma (CCA) and normal serum samples based on different biomarkers. The metabolic data were visualized using a heatmap, with red indicating higher concentrations of specific metabolites and blue representing lower concentrations. The tumor group (CCA) and the control group (normal serum) exhibit significant metabolic differences, particularly in lipid metabolism (e.g., LysoPC, Glycocholic acid) and amino acid metabolism (e.g., Sphinganine, 2-Hydroxybutyric acid). CCA: cholangiocarcinoma.

The heatmap (Figure 3) displays the relative concentrations of various biomarkers (e.g., LysoPC, Sphinganine, Glycocholic acid) in both groups. Red indicates higher concentration levels, while blue indicates lower concentration levels. Compared with the control group, 15 metabolites in the CCA group showed significant increases, including most LysoPCs and five organic acids: glycohyocholic acid, D-glucuronic acid, 2-hydroxybutyric acid, 2-hydroxy-3-methylbutyric acid, and ureidoisobutyric acid. In contrast, 10 metabolites were significantly decreased in the CCA group, including the remaining LysoPCs, phosphatidylcholines (PCs), sphinganine, ketoleucine, and two organic acids, alpha-ketoisovaleric acid and glyceric acid. The comparison of the MS signal intensities for each marker metabolite is presented in Figure 3.

**FIGURE 4:**
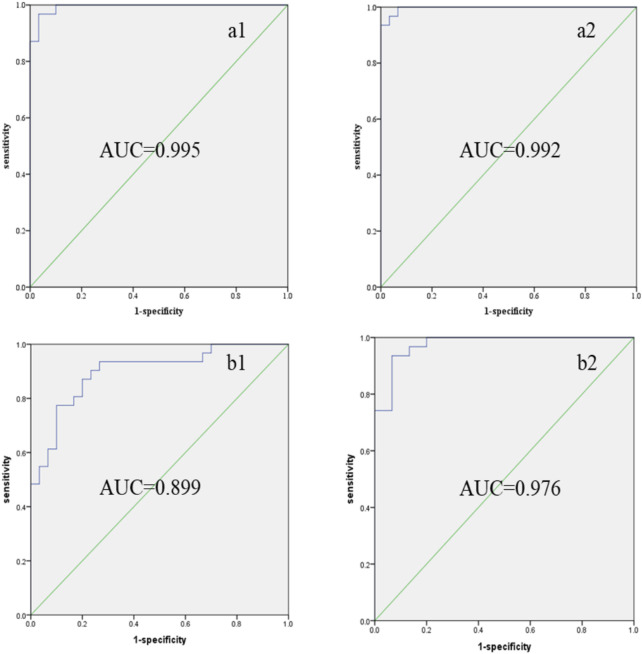
Combined indexes: ROC curves of differential marker metabolites in CCA patients’ serum. **(a1)** Increased levels of biomarkers (MS positive mode); **(a2)** decreased levels of biomarkers (MS positive mode); **(b1)** increased levels of biomarkers (MS negative mode); **(b2)** decreased levels of biomarkers (MS positive mode). ROC: receiver operating characteristic; AUC: area under the curve.

To further validate the accuracy of the differential metabolites, a combined-index ROC curve analysis was conducted, which provides a more precise demonstration of the impact of statistical metabolites on CCA. As shown in Figure 4, the AUC for increased and decreased biomarkers in positive mode was 0.995 and 0.992, respectively, while the AUC for increased and decreased biomarkers in negative mode was 0.899 and 0.976, respectively. It was observed that, except for the metabolites with reduced content (identified in the negative mode), which exhibited a certain accuracy (AUC in the range of 0.7–0.9), all other screened metabolites showed higher diagnostic accuracy for CCA (AUC >0.9). These results indicate that the ROC analysis demonstrated a satisfactory accuracy for the metabolites identified in our study.

**FIGURE 5:**
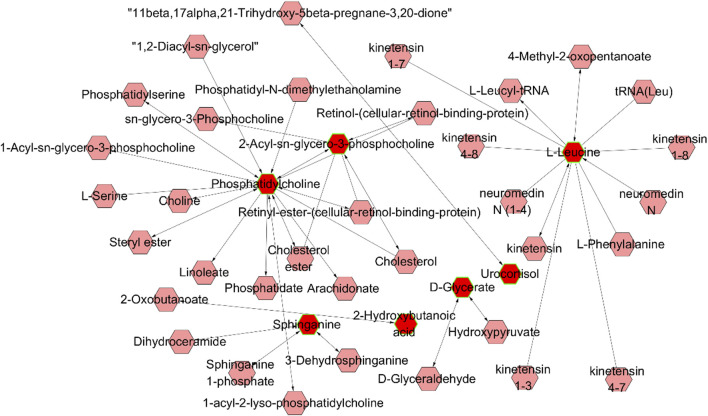
Global network of the remarkably perturbed metabolic pathways in CCA by MetScape analysis. Red hexagons represent the identified differentially expressed metabolites, and pink hexagons were the involved metabolites that have not been identified in this study.

To further elucidate the biological functions of these altered metabolites, a systematic metabolic pathway analysis was performed using KEGG pathway enrichment and topological analysis based on pathway impact values (*P*-values). A total of seven pathways were identified, including the phosphatidylinositol signaling system, sphingolipid metabolism, L-leucine metabolism, D-glycerate metabolism, and glycerophospholipid metabolism (Figure 5).

## 4 Discussion

Metabolic reprogramming emerges as a central driver of cholangiocarcinoma (CCA) progression through dynamic crosstalk within the tumor microenvironment (TME). Our integrated metabolomic and functional analyses reveal that TME remodeling in CCA is orchestrated by multifaceted metabolite interactions. Complementing these findings, phospholipid dysregulation—evidenced by reduced phosphatidylcholine (PC) and elevated lysophosphatidylcholine (LysoPC)—promotes carcinogenic ROS/DNA damage and aberrant lysophosphatidic acid (LPA) signaling, while perturbations in bile acid, sphingolipid, and amino acid metabolism (e.g., glycohyocholic acid accumulation, sphingosine depletion, ketoleucine reduction) collectively underscore CCA’s reliance on metabolic rewiring for immune evasion and proliferation. These insights align with the hallmarks of cancer framework and highlight actionable nodes for therapeutic intervention targeting metabolic vulnerabilities in CCA.

### 4.1 Phospholipid metabolism

Cell proliferation is a fundamental requirement for tumorigenesis, which in turn necessitates increased metabolic activities such as elevated glycolysis and lipid synthesis (DeBerardinis et al., 2008). Phosphatidylcholine (PC) is the most abundant phospholipid in mammalian cells, comprising approximately 40%–50% of the total cellular phospholipids (van der Veen et al., 2017). PC predominantly resides in the outer leaflet of the cell membrane (Devaux, 1991). Changes in PC levels are associated with various liver diseases, including nonalcoholic fatty liver disease (Maev et al., 2020), liver failure (Li et al., 2021), and liver cancer (Cotte et al., 2019). LysoPC, a downstream product of PC, is also implicated in cancer progression and recurrence (Banales et al., 2020). In bile duct cells, LysoPC influences the expression of *β*-galactosidase, a marker of cellular senescence, enhances reactive oxygen species production, and induces DNA damage, which may lead to carcinogenesis (Shimizu et al., 2015). Furthermore, LysoPC has been identified as a biomarker for several cancers. For example, lower levels of LysoPC(16:0/0:0) have been observed in ovarian cancer (Kim et al., 2014) and colorectal cancer (Zhao et al., 2007). LysoPC (16:0/0:0) has also been used as a biomarker for intrahepatic CCA (Kim et al., 2017). In our study, we observed that serum levels of PC were lower in CCA patients compared to the normal group, while LysoPC levels showed a marked increase (Figure 3). The liver plays a crucial role in maintaining lipid and lipoprotein homeostasis. CCA disrupts hepatic lipid metabolism, while cancer cells require an abundant supply of lipids for proliferation, resulting in a decrease in serum PC levels. Conversely, LysoPC is generated through the hydrolysis of PC by phospholipase A_2_. Normal LysoPC metabolism produces lysophosphatidic acid (LPA), a potent cellular signaling molecule that acts as a strong mitogen. Thus, the altered LysoPC metabolism observed in the serum of CCA patients reflects an abnormal lipid metabolism associated with the disease.

### 4.2 Bile acid metabolism

Bile acids are critical biomolecules involved in vertebrate metabolism, contributing to processes such as the regulation of cholesterol homeostasis (Dalton et al., 2021), promotion of lipid and fat-soluble vitamin metabolism (Di Ciaula et al., 2017), and antibacterial defense (Park et al., 2021). Given their involvement in key signaling pathways regulating cell proliferation and apoptosis (Duboc et al., 2014), elevated bile acid levels have been linked to CCA (Neale et al., 1971), and an increase in serum bile acid concentrations is commonly associated with liver cancer (Thomas et al., 2021). In this study, we observed elevated levels of glycohyocholic acid, which may be indicative of CCA-induced disruption of hepatic bile acid homeostasis. Although increased bile acid concentrations are also seen in other liver diseases, glycohyocholic acid may not serve as a specific biomarker for CCA due to its broader association with various liver conditions.

### 4.3 Sphingomyelin metabolism

Sphingomyelin is a major component of the cell membrane and plays a vital role in cell growth, senescence, and apoptosis (Hori et al., 2021). Sphingosine, a product of sphingolipid metabolism, is converted into sphingosine-1-phosphate (S1P) through phosphorylation. S1P has been shown to be involved in a wide array of physiological processes, including cell proliferation, differentiation, and apoptosis (Li et al., 2015). Additionally, sphingosine has been implicated in the regulation of various pathological processes, including inflammation and cancer (Nagahashi et al., 2018). The abnormal metabolism of sphingosine in the liver is frequently associated with the progression of liver cancer (Xie et al., 2017), owing to the pivotal role of sphingomyelin in hepatocyte lipid metabolism (Miura et al., 2021). However, the exact mechanisms through which sphingosine contributes to cancer development remain unclear. Nonetheless, Uranbileg et al. reported that sphingosine kinase (SK), the enzyme responsible for degrading sphingosine, plays a crucial role in the proliferation and migration of cancer cells, leading to a reduction in serum sphingosine levels in cancer patients (Uranbileg et al., 2016). Our study also found that serum sphingosine levels were decreased in CCA patients, consistent with the findings in previous reports.

### 4.4 Amino acids and other metabolites

Amino acids serve as the building blocks for protein synthesis and are integral to cancer metabolism (Lieu et al., 2020). The literature suggests that branched-chain amino acids may play a role in the treatment of advanced HCC (O’Connell, 2013). Leucine, in particular, has been shown to influence cell growth and signaling pathways in HCC cell lines and is used as a supplement to inhibit tumor cell proliferation (Hassan et al., 2021). In our study, serum metabolite analysis revealed abnormal metabolism of several organic acids and amino acids in CCA patients. Notably, a reduction in ketoleucine levels in the serum of CCA patients was linked to tumor cell proliferation. D-glucuronic acid, which can be converted by chondroitin–glucuronate C5-epimerase—a tumor rejection antigen expressed in various cancer tissues (Mizukoshi et al., 2012)—was found to be elevated in CCA patients’ serum due to increased expression of this enzyme. Disruptions in the metabolism of branched-chain fatty acids, metabolites of amino acids, have also been observed in lung cancer studies (Zablocka-Slowinska et al., 2018). In this work, increased serum levels of 2-hydroxybutyric acid in CCA patients, compared with the healthy group, may contribute to oxidative stress in these patients (Gall et al., 2010). This increase in 2-hydroxybutyric acid could be a by-product of enhanced hepatic glutathione synthesis, a response to oxidative stress (Zeng et al., 2014). Additionally, glyceric acid, an intermediate in serine degradation, was found to be reduced in the serum of CCA patients. Glyceric acid is phosphorylated to form 3-phosphoglycerate, which plays a critical role in glycolysis, an important energy pathway for tumor cells (Jiang et al., 2018). Consistent with this, reduced glyceric acid levels have been reported in the blood of breast cancer patients (Nishiumi et al., 2010). Furthermore, ureidoisobutyric acid, typically associated with pyrimidine metabolism, was found to be abnormally elevated in the serum of CCA patients. The continuous supply of pyrimidines is essential for cancer cell survival (Siddiqui and Ceppi, 2020), and the observed increase in ureidoisobutyric acid reflects the growing demand for pyrimidines in proliferating cancer cells.

## 5 Conclusion

In this study, an unbiased metabolomics approach using UPLC-Q-TOF-MS was employed to identify serum metabolites in CCA patients and healthy controls. Through statistical analysis, 25 marker metabolites with significant alterations were identified in the serum of CCA patients. Of these, 15 metabolites were significantly elevated, while 10 were significantly decreased. Notably, metabolic disruptions were observed in glycerophospholipid metabolism, sphingolipid metabolism, and L-leucine metabolism pathways, among others. These metabolites could effectively differentiate CCA patients from healthy individuals. The AUC for increased and decreased biomarkers in positive mode were 0.995 and 0.992, respectively, while the AUC for increased and decreased biomarkers in negative mode were 0.899 and 0.976. These findings highlight the potential of these metabolites as diagnostic biomarkers for CCA. This study serves as a foundation for further research, encouraging the collection of additional samples and comparisons of serum metabolites across different patient groups to identify and validate specific biomarkers for CCA.

## Data Availability

The original contributions presented in the study are included in the article/Supplementary Material, further inquiries can be directed to the corresponding authors.
