# Supplementary material for: UPLC-Q-TOF-MS-based unbiased serum metabolomics investigation of cholangiocarcinoma
Source: Front Mol Biosci. 2025 Apr 7;12:1549223. doi: 10.3389/fmolb.2025.1549223 (PMC12009706; doi:10.3389/fmolb.2025.1549223)
Supplement: Supplementary file 1 [file Table1.docx]

**Supplementary Table 1** Mobile phase gradient program

| Time/min | Mobile phase A (%) | Mobile phase B (%) |
| --- | --- | --- |
| 0 | 99 | 1 |
| 5 | 75 | 25 |
| 9 | 50 | 50 |
| 9.1 | 1 | 99 |
| 11 | 1 | 99 |
| 11.1 | 99 | 1 |
| 13 | 99 | 1 |

| Pathway | *P* Value | Impact |
| --- | --- | --- |
| Sphinganine | 5.34E-10 | 3.53 |
| D-Glycerate | 2.58E-5 | 1.79 |
| 2-Acyl-sn-glycero-3-phosphocholine | 0.00191 | 7.31 |
| Urocortisol | 0.0112 | 2.41 |
| 2-Hydroxybutanoic acid | 0.0203 | 2.94 |
| Phosphatidylcholine | 0.0221 | 1.41 |
| L-Leucine | 0.0285 | 1.53 |

**Supplementary Table 2**The impact values and p-values of the seven metabolic pathways
